# Dichotomous Roles of Programmed Cell Death 1 on HIV-Specific CXCR5^+^ and CXCR5^−^ CD8^+^ T Cells during Chronic HIV Infection

**DOI:** 10.3389/fimmu.2017.01786

**Published:** 2017-12-12

**Authors:** Yan-Mei Jiao, Hong-Ge Yang, Hui-Huang Huang, Bo Tu, Shao-Jun Xing, Lin Mao, Wei Xia, Ran He, Ji-Yuan Zhang, Ruo-Nan Xu, Lei Jin, Ming Shi, Zhe Xu, En-Qiang Qin, Xi-Cheng Wang, Hao Wu, Lilin Ye, Fu-Sheng Wang

**Affiliations:** ^1^Treatment and Research Center for Infectious Diseases, Beijing 302 Hospital, Beijing, China; ^2^Savaid Medical School, University of Chinese Academy of Sciences, Beijing, China; ^3^Department of Microbiology, Carver College of Medicine, University of Iowa, Iowa City, IA, United States; ^4^Yunnan Provincial Hospital of Infectious Diseases, Kunming, China; ^5^Center for Infectious Diseases, Beijing You’an Hospital, Capital Medical University, Beijing, China; ^6^Institute of Immunology, Third Military Medical University, Chongqing, China

**Keywords:** HIV, CXCR5^+^CD8^+^ T cells, programmed cell death 1, cytotoxic T cells, CXCR5^−^CD8^−^ T cells

## Abstract

**Background:**

CXCR5^+^CD8^+^ T cells have been demonstrated to play an important role in the control of chronic viral replication; however, the relationship between CXCR5^+^CD8^+^ T cells, HIV disease progression, and programmed cell death 1 (PD-1) expression profile on CXCR5^+^CD8^+^ T cells during HIV infection remain poorly understood.

**Methods:**

We enrolled a total of 101 HIV patients, including 62 typical progressors, 26 complete responders (CRs), and 13 immune non-responders (INRs). Flow cytometric analysis, immunohistochemical staining, and relative function (i.e., cytokine secretion and PD-1 blockade) assays were performed to analyze the properties of CXCR5^+^CD8^+^ T cells.

**Results:**

HIV-specific CXCR5^+^CD8^+^ T cells in the peripheral blood and distribution of CXCR5^+^CD8^+^ T cells in the lymph node (LN) were negatively correlated with disease progression during chronic HIV infection. PD-1 was highly expressed on CXCR5^+^CD8^+^ T cells and positively associated with peripheral CD4^+^ T cell counts. Functionally, IFN-γ and TNF-α production of CXCR5^+^CD8^+^ T cells were reduced by PD-1 pathway blockade, but the production of IFN-γ and TNF-α from CXCR5^−^CD8^+^ T cells increased in response to TCR stimulation. Interestingly, PD-1 expression was constantly retained on CXCR5^+^CD8^+^ T cells while significantly decreased on CXCR5^−^CD8^+^ T cells after successful antiretroviral treatment in chronic HIV-infected patients.

**Conclusion:**

PD-1^+^CXCR5^+^CD8^+^ T cells are functional cytotoxic T cells during chronic HIV infection. PD-1^+^CXCR5^+^CD8^+^ T cells may represent a novel therapeutic strategy for the disease.

## Introduction

HIV-specific CD8^+^ T cells play a critical role in controlling HIV replication and there is direct relationship between HIV-specific CD8^+^ T cells and HIV control (1, 2). Emerging data suggest that if HIV-specific CD8^+^ T cells can be effectively harnessed, HIV could be eliminated (3–5). Although HIV-specific CD8^+^ T cells showed an exhausted phenotype to a certain extent during chronic HIV infection, they can still inhibit viral replication. Some studies have shown that a subset of CD8^+^ T cells expressing the chemokine receptor CXCR5 share some features of HIV-specific CD8^+^ T cells and play a pivotal role in the control of viral replication during chronic viral infections (6–8). In addition, it was reported that CXCR5^+^CD8^+^ T cells exhibit a more potent proinflammatory function than CXCR5^−^CD8^+^ T cells during chronic HIV infections (6, 9). Despite these findings, the role of CXCR5^+^CD8^+^ T cells in the context of a chronic HIV infection, as well as the effect of ART on CXCR5^+^CD8^+^ T cells require further clarification.

One of the important characteristics of a chronic HIV infection is CD8^+^ T cell dysfunction associated with the expression of the programmed cell death 1 (PD-1) inhibitory receptor (10–13). PD-1 is a central regulator of CD8^+^ T cell exhaustion, and blockade of the PD-1 pathway has a beneficial effect on enhancing T cell immunity in chronic viral infections (10–12, 14–17). It has also been reported that a blockade of the PD-1 pathway did not completely restore T cell function (18–20); this means that other mechanisms impacting CD8^+^ T cell functionality may exist. There are different opinions regarding the expression of PD-1 on CXCR5^+^CD8^+^ T cells during chronic viral infections. He et al. (6) reported lower levels of PD-1 expression on CXCR5^+^CD8^+^ T cells compared to CXCR5^−^CD8^+^ T cells during chronic viral infection, whereas other researchers (8, 9, 21, 22) reported a higher PD-1 expression on CXCR5^+^CD8^+^ T cells. CXCR5^+^CD8^+^ T cells share multiple characteristics with follicular helper T cells (TFHs; CXCR5^+^CD4^+^ T cells) (6, 8, 9, 21). PD-1 is highly expressed on TFHs and is a critical functional molecule for TFHs (23–25). Whether PD-1 is also a critical functional molecule for CXCR5^+^CD8^+^ T cells during chronic HIV infection remains unknown.

In this study, we enrolled HIV-infected typical progressors (TPs), antiviral therapy complete responders (CRs) and immune non-responders (INRs), and analyzed the CXCR5^+^CD8^+^ T cells from blood and lymphoid tissue specimens. The data showed that CXCR5^+^CD8^+^ T cells were negatively associated with HIV disease progression during chronic HIV infection. CXCR5^+^CD8^+^ T cells exhibited different PD-1 expression profile and response to PD-1 blockade compared to CXCR5^−^CD8^+^ T cells. Thus, PD-1^+^CXCR5^+^CD8^+^ T cells can be classified as functional cytotoxic T cells (CTLs) during chronic HIV infection.

## Materials and Methods

### Subjects

All samples were collected with the approval of the Beijing 302 Hospital Research Ethnics Committee and were written informed consent in accordance with the Declaration of Helsinki. The methods were carried out in accordance with approved guidelines and regulations. Peripheral blood for isolation of peripheral blood mononuclear cells (PBMCs) were obtained from HIV-infected patients. Lymph node (LN) biopsies were collected from nine treatment naïve patients. HIV status was defined according to previous reports (26, 27). Patients included a cohort of 62 TPs (who exhibited a typical progressive disease without receiving antiviral treatment), 26 CRs to antiviral therapy for more than 2 years with peripheral CD4^+^ T cell counts above 350 cells/μL and plasma HIV-1 RNA <80 copies/mL, and 13 INRs to antiviral therapy for more than 2 years with CD4^+^ T cell counts below 200 cells/μL and plasma HIV-1 RNA <80 copies/mL. Exclusion criteria included coinfection with HBV, HCV, tuberculosis, pregnancy, and moribund status (28). The detailed information for the donors is listed in Table 1.

**Table 1:** Subject characteristics.

	Typical progressors (TPs) (cells/μL)			CRs	INRs
	CD4 ≤ 200	200 < CD4 ≤ 350	CD4 > 350		
Cases (*n*)	17	25	20	26	13
Age (years)	30 (19–62)	32 (22–62)	26 (20–36)	34 (21–55)	35 (22–51)
Gender (M/F)	17/0	25/0	20/0	26/0	13/0
CD4^+^ T (cells/μL)	152 (25–190)	270 (205–341)	441 (361–813)	456 (352–727)	140 (35–197)
Viral load (copies/mL)	77,371 (16,514–1,210,000)	44,373 (5,032–333,000)	20,990 (2,541–111,615)	<80	<80

*Data are expressed as the median (range), unless otherwise stated*.

*CRs, complete responders; INRs, immune non-responders; M, male; F, female*.

### Plasma HIV-1 RNA Monitoring

The HIV-1 RT-PCR Assay V2 (QIAGEN, Hilden, Germany) and CFX96 Real-Time System (Bio Rad Laboratories, Hercules, CA, USA) were used to quantify the HIV-1 RNA levels in plasma as previously described (11, 29). The cut-off value was 80 copies/mL.

### Flow Cytometry

For phenotypic staining, PBMCs and milled LN cells were extracellularly stained using antibodies specific to respective markers, including anti-CD3-PerCP (BD Biosciences, Franklin Lakes, NJ, USA), anti-CD8-FITC (eBioscience, Waltham, MA, USA), anti-CXCR5-eFlour450 (eBioscience, Waltham, MA, USA), anti-PD-1-BV500 (BD Biosciences, Franklin Lakes, NJ, USA), and HIV Pentamer-PE (HIV-1 gag p17 76-84R, HIV-1 gag gp41 67-75R, and HIV-1 nef 72-82R) (Proimmune, Oxford, UK) for 30 min at room temperature. The cells were washed with FACS buffer, and assessed by flow cytometry (6). After extracellular staining, the cells were permeabilized, fixed and stained using the Permeabilization/Fixation Kit according to manufacturer’s instructions (eBioscience, Waltham, MA, USA). The cells were then incubated for 30 min at 4°C with antibodies specific to granzyme B-FITC (BD Biosciences, Franklin Lakes, NJ, USA) and perforin-Alexa647 (BD Biosciences, Franklin Lakes, NJ, USA).

After stimulated by overlapping peptides covering the HIV-1 pol, gag, and env antigens (JPT, Berlin, Germany) for 8 hrs with or without PD-L1(10 µg/mL) in the presence of brefeldin A and CD107a-eFluor660 (eBioscience, Waltham, MA, USA), extracellular stained, and permeabilized, the PBMCs or sorted cells were then incubated for 30 min at 4°C with antibodies specific to IFN-γ-eFlour506 (eBioscience, Waltham, MA, USA) and TNF-α-PE-Cy7 (eBioscience, Waltham, MA, USA). Flow cytometric acquisition was performed on a FACSVerse or Caliber.

### Immunohistochemistry and Confocal Microscopy

Paraffin-embedded sections of acetone-fixed LN biopsies were incubated with anti-CD8, anti-CXCR5, and anti-CD20 antibodies overnight at 4°C after the endogenous peroxidase activity was blocked with 0.3% H_2_O_2_. 3-amino-9-ethyl-carbazole (red color) was used as the substrate followed by counterstaining with hematoxylin for single staining according to previously described protocols (30–32). Images (100×, 400×) were acquired with an Olympus CX31 microscope and Olympus FV1000 confocal microscope.

### Cell Sorting

Positive selection was adopted for obtaining CD8^+^ T cells from PBMCs using the MiniMACS system (Miltenyi Biotech, Bergisch-Gladbach, Germany) according to the manufacturer’s instructions. CD8^+^ T cells were then stained with anti-CD8-PE-Cy7 (BD Biosciences, Franklin Lakes, NJ, USA) and anti-CXCR5-Alexa Flour 488 (BD Biosciences, Franklin Lakes, NJ, USA) for 30 min at room temperature and sorted using a FACSAriaII. The purity of the sorted cells was >95% for all sorting experiments.

### Killing Assay

To investigate the killing capacity of CXCR5^+^CD8^+^ T and CXCR5^−^CD8^+^ T cells, we co-cultured PBMCs, purified CXCR5^+^CD8^+^ T cells or CXCR5^−^CD8^+^ T cells with Jurkat cells [HIV-infected CD4^+^ T cells (33)] integration of the HIV cDNA stimulated by overlapping peptides covering the HIV-1 pol, gag, and env antigens (JPT, Berlin, Germany). The total cells were stimulated by the peptide pools (1 µg/mL, 100 µL per sample) and brefeldin A for 8 h at 37°C in the presence of 5% CO_2_ before conducting surface and intracellular staining (6). To evaluate the level of apoptosis, the cells were washed with FACS buffer and stained with CD4-APC (eBioscience, Waltham, MA, USA), 7-AAD-PerCP (BD Biosciences, Franklin Lakes, NJ, USA) and Annexin V-PE (Southern Biotech, Birmingham, AL, USA). Flow cytometric acquisition was performed on a FACSVerse or Caliber.

### Statistical Analysis

All data were analyzed using SPSS version 22. A non-parametric Kruskal–Wallis test was used for multiple comparisons among different groups, and a Mann–Whitney *U* test was used for the comparison between two groups. A paired Student’s *t-*test was adopted for the analysis with or without PD-L1. The correlations between variables were evaluated by a Spearman rank correlation test. For all tests, *P* values <0.05 indicated a significant difference (28).

## Results

### HIV-Specific CXCR5^+^CD8^+^ T Cells Were Negatively Correlated with Disease Progression during Chronic HIV Infection

**Figure 1:**
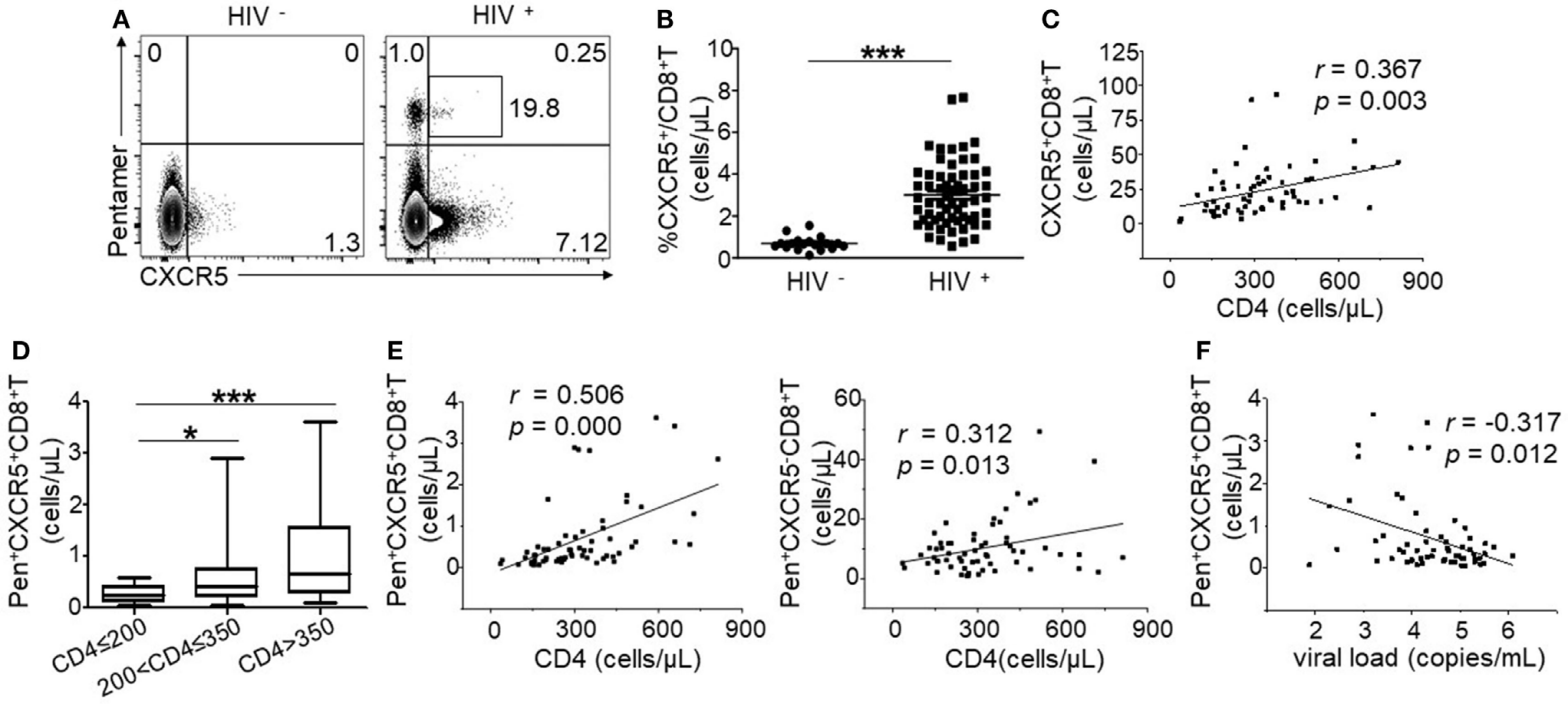
HIV-specific CXCR5^+^CD8^+^ T cells are negatively correlated with disease progression during chronic HIV infection. **(A)** Representative flow cytometric data of CXCR5 and Pentamer staining from the peripheral blood of healthy controls and HIV-infected patients (gated on CD3^+^CD8^+^ lymphocytes). The values in the quadrant represent the frequency of individual populations. The number in the box represents the frequency of CXCR5^+^ expression among the Pentamer^+^ population. **(B)** Comparison of the percentage of CXCR5^+^CD8^+^ T cells between the healthy controls and typical progressers. **(C)** Positive correlation between peripheral CXCR5^+^CD8^+^ T cells and CD4^+^ T cell counts (*p* = 0.003). **(D)** Statistical analysis of the number of CD3^+^CD8^+^Pentamer^+^CXCR5^+^ T cells in individual groups with different CD4^+^ T cell numbers. Multiple comparisons between the different groups were made using a Kruskal–Wallis H non-parametric test (*p < 0.05, ***p < 0.001). **(E)** Positive correlation between the number of CD3^+^CD8^+^Pentamer^+^CXCR5^+^ T cells (left, *p* = 0.0001), CD3^+^CD8^+^Pentamer^+^CXCR5^−^ T cells (right, *p* = 0.0013) and CD4^+^ T cell counts. **(F)** Negative correlation between the absolute cell number of CD3^+^CD8^+^Pentamer^+^CXCR5^+^ T cells and plasma viral load. Each dot represents one individual. The correlation analysis was evaluated *via* a Spearman rank correlation test. Solid line, linear growth trend; *r*, correlative coefficient. *p* values are shown.

To investigate circulating CXCR5^+^CD8^+^ T cells, we first detected the frequency of total and HIV-specific CXCR5^+^CD8^+^ T cells. There was a small population of CXCR5^+^CD8^+^ T cells (Figures 1A,B) in healthy controls. The frequency of total CXCR5^+^CD8^+^ T cells was obviously increased in the HIV-infected patients compared with the healthy controls (Figures 1A,B). Among the Pentamer^+^ CTLs, we clearly identified one population of CXCR5^+^CD8^+^ T cells, indicating that chronic HIV infection can induce HIV-specific CXCR5^+^CD8^+^ T cells. A correlation analysis demonstrated that there was a positive correlation between CXCR5^+^CD8^+^ T cells and peripheral CD4^+^ T cell counts (Figure 1C; *r* = 0.367; *p* = 0.003). Furthermore, the increased amount of HIV-specific CXCR5^+^CD8^+^ T cells was associated with higher CD4^+^ T cell counts (Figures 1D,E left), with better correlation coefficient and significance probability than that of CXCR5^−^CD8^+^ T cells (Figure 1E right). In addition, the absolute counts (Figure 1F) of peripheral HIV-specific CXCR5^+^CD8^+^ T cells were inversely correlated with the viral load. Thus, these data suggest that chronic HIV infection induced CXCR5^+^CD8^+^ T cells, which are associated with disease progression during chronic HIV infection.

### CXCR5^+^CD8^+^ T Cells in LN Correlated with CD4^+^ T Cell Counts

**Figure 2:**
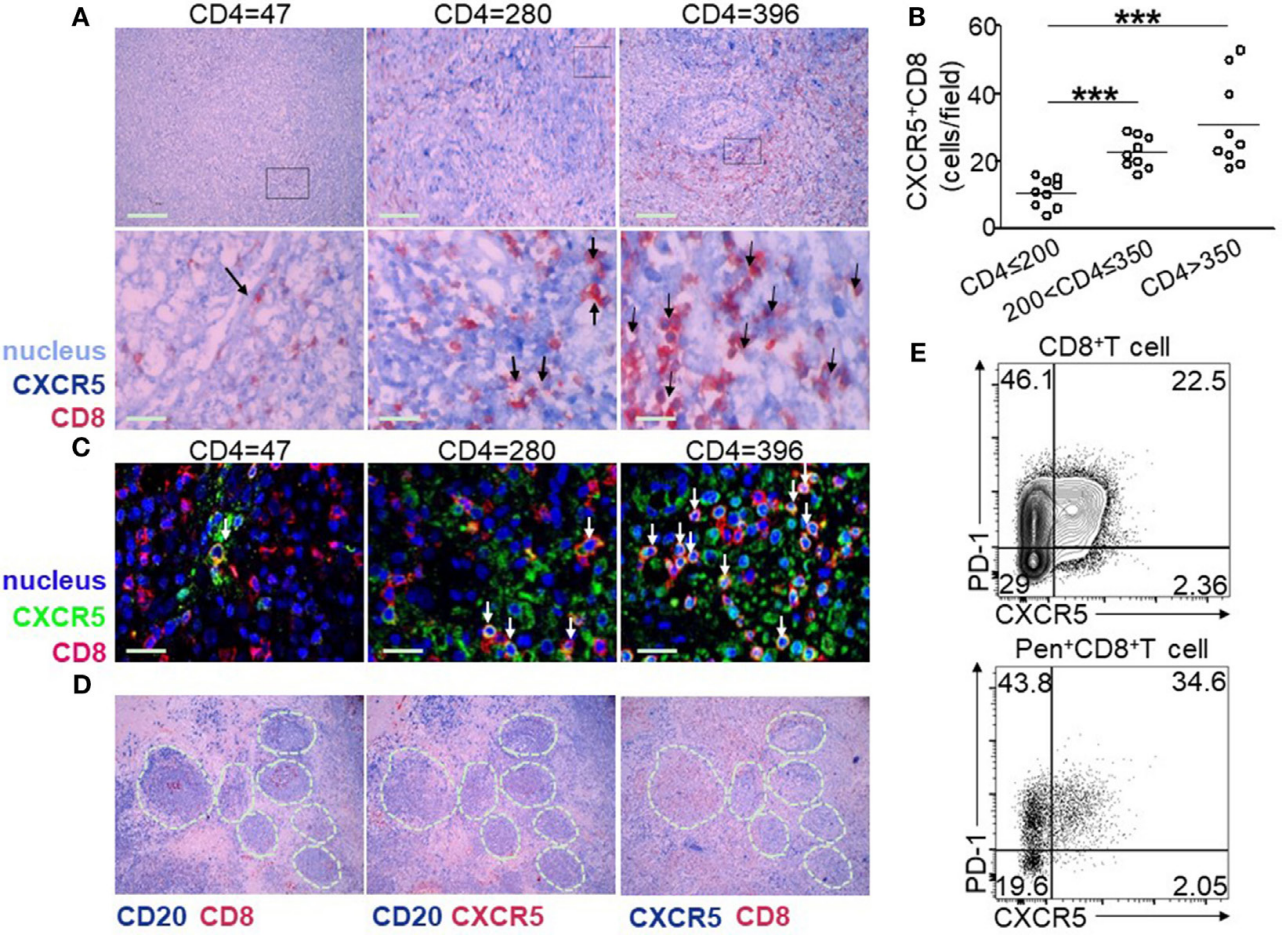
Lymph node (LN) CXCR5^+^CD8^+^ T cells are associated with peripheral CD4^+^ T cell counts. **(A)** Representative immunohistochemical data show the tissue localization of CXCR5^+^ (dark blue) CD8^+^ (red) T cells (dark arrow) in the LNs from nine HIV-infected patients with different CD4^+^ T cell counts. The cell nuclei are stained light blue with hematoxylin. **(B)** Confocal microscopy of lymph nodes (LNs) stained with CXCR5^+^ (green) and CD8^+^ (red). The nuclei are stained using DAPI (blue). CXCR5^+^CD8^+^ cells are double stained in yellow (white arrow). **(C)** Statistical analysis of CXCR5^+^CD8^+^ T cells from three different patient groups. Each dot indicates one LN from one individual patient. The data represent three independent experiments with similar results (*n* > 3 for each group, Kruskal–Wallis H non-parametric test ***p < 0.001). **(D)** Immunohistochemical data of the same tissue of LNs with different markers: left-CD20 (dark blue) and CD8 (red), middle-CD20 (dark blue) and CXCR5 (red), and right-CXCR5 (dark blue) and CD8 (red). CD20^+^ cells are used for the identification of germinal centers (GCs; white dashed lines indicate GCs). **(E)** Representative flow cytometric data of programmed cell death 1 (PD-1) and CXCR5 staining gated on total CD8^+^ T cells and Pentamer^+^CD8^+^ T cells from the cells of LN.

To visualize CXCR5^+^CD8^+^ T cells in the LN, immunohistochemical staining was performed using antibodies against CXCR5, CD8, and CD20. Double-positive staining of CXCR5 (dark blue) and CD8 (red) was defined as CXCR5^+^CD8^+^ T cells, CD20 was used for the identification of germinal center (GC). As shown in Figure 2A, the LNs from HIV-infected patients with low CD4^+^ T cell counts (<200 cells/μL) exhibited an impaired lymphoid structure, including broken lymphoid follicles, few CD8^+^ T cells, and enhanced tissue fibrosis. Moreover, few CXCR5^+^CD8^+^ T cells were found (Figure 2A left). By contrast, in the LNs from HIV-infected patients with CD4^+^ T cell counts above 200 cells/μL, the lymphoid structure remained relatively intact, accompanied by normal lymphoid follicles and lymphocyte distribution (Figure 2A middle and right). There were more CXCR5^+^CD8^+^ T cells distributed in the LNs with higher CD4^+^ T cell counts by quantitative analysis (Figure 2B). In addition, confocal images confirmed CXCR5 and CD8 double staining of T cells and the enhanced distribution of CXCR5^+^CD8^+^ T cells in the LNs from patients with higher CD4^+^ T cell counts (Figure 2C). Both CD8 (Figure 2D left) and CXCR5 (Figure 2D middle) can be found in GCs, and also CXCR5^+^CD8^+^ T cells were localized in and out of GCs (Figure 2D right). Thus, consistent with the peripheral lymphocytes, patients of higher CD4^+^ T cell counts exhibited more CXCR5^+^CD8^+^ T cells residing in the LN, where CXCR5^+^CD8^+^ T cells can be found in and out of GCs. One integrated LN and the relevant mononuclear cell was gotten, and the results of flow analysis showed that there were higher PD-1 expression on CXCR5^+^ T cells and HIV-specific CXCR5^+^ T cells than that of CXCR5^−^ T cells (Figure 2E).

### PD-1 Was Highly Expressed on CXCR5^+^CD8^+^ T Cells and Negatively Associated with HIV Disease Progression

**Figure 3:**
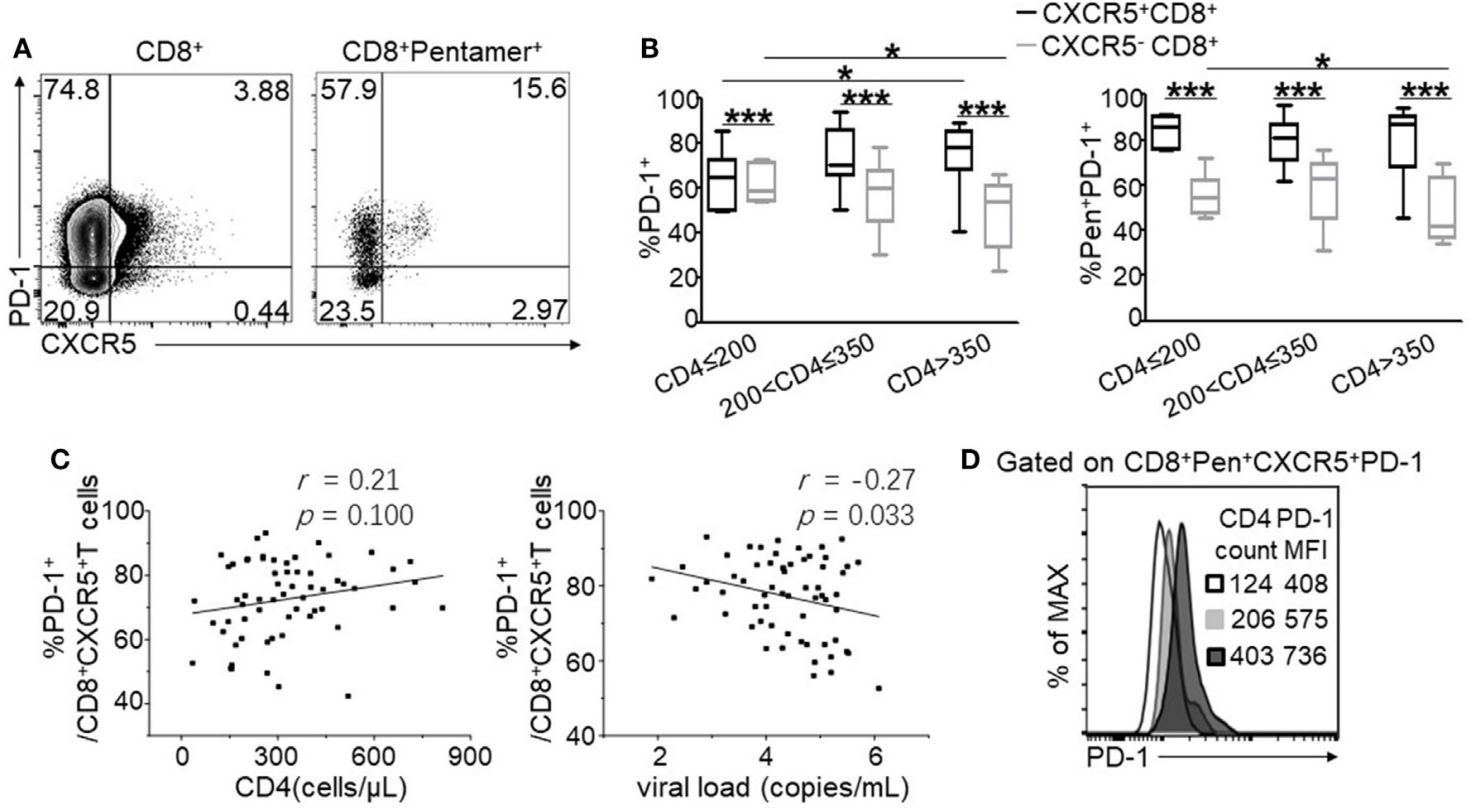
Programmed cell death 1 (PD-1) is highly expressed on CXCR5^+^CD8^+^ T cells and negatively associated with HIV disease progression. **(A)** Representative flow cytometric data of PD-1 and CXCR5 staining gated on total CD8^+^ T cells and Pentamer^+^CD8^+^ T cells, respectively, from the peripheral blood. **(B)** Statistical analysis of PD-1 expression on CXCR5^+^ and CXCR5^−^CD8^+^ T cells in three groups categorized by CD4^+^ T cell counts. **(C)** Correlation between the percentage of CD3^+^CD8 ^+^CXCR5^+^ PD-1^+^ T cells and CD4^+^ T cell counts (left, *p* = 0.100) or viral load (right, *p* = 0.033). **(D)** Representative data of PD-1 mean fluorescence intensity (MFI) on Pentamer^+^CXCR5^+^CD8^+^ T cells. The data represent three independent experiments with similar results (*n* > 5 for each group).

Programmed cell death 1 is an important functional marker highly expressed on CD4^+^ follicular T cells; however, the function of PD-1 on CXCR5^+^CD8^+^ T cells remains controversial (7, 34). Thus, we analyzed the expression of PD-1 on the total and HIV-specific CXCR5^+^CD8^+^ T cells. Compared with CXCR5^−^CD8^+^ T cells, CXCR5^+^CD8^+^ T cells exhibited enhanced PD-1 expression (Figure 3A left), which was more evident in HIV-specific CTLs (Figure 3A right). Statistically analysis demonstrated that PD-1 exhibited a higher expression on CXCR5^+^CD8^+^ T cells compared to CXCR5^−^CD8^+^ T cells at various stages of disease progression (Figure 3B) for both the total and HIV-specific CTLs. Interestingly, PD-1 expression showed an exact opposite pattern on CXCR5^−^ and CXCR5^+^CD8^+^ T cells during the disease progression. CXCR5^−^CD8^+^ T cells showed significantly lower PD-1 expression in patients with CD4^+^ T cell counts more than 350 cells/μL for both the total and Pentamer^+^CD8^+^ T cells (Figure 3B; *p* < 0.05), whereas CXCR5^+^CD8^+^ T cells exhibited elevated PD-1 expression in patients with higher CD4^+^ T cell counts (Figure 3B; *p* < 0.05). The higher frequency of PD-1^+^CXCR5^+^CD8^+^ T cells was associated with lower viral load (*r* = −0.27, *p* = 0.033; Figure 3C right), while there were tendency positively correlation between the frequency of PD-1^+^CXCR5^+^CD8^+^ T cells and CD4^+^ T cell counts (*r* = 0.21, *p* = 0.100; Figure 3C left). In addition, the mean fluorescence intensity (MFI) of PD-1 expression on HIV-specific CXCR5^+^CD8^+^ T cells increased with the elevation of peripheral CD4^+^ T cell counts (Figure 3D).

### PD-1 Pathway Blockade Reduced IFN-γ and TNF-α Production of CXCR5^+^CD8^+^ T Cells in Response to TCR Stimulation

**Figure 4:**
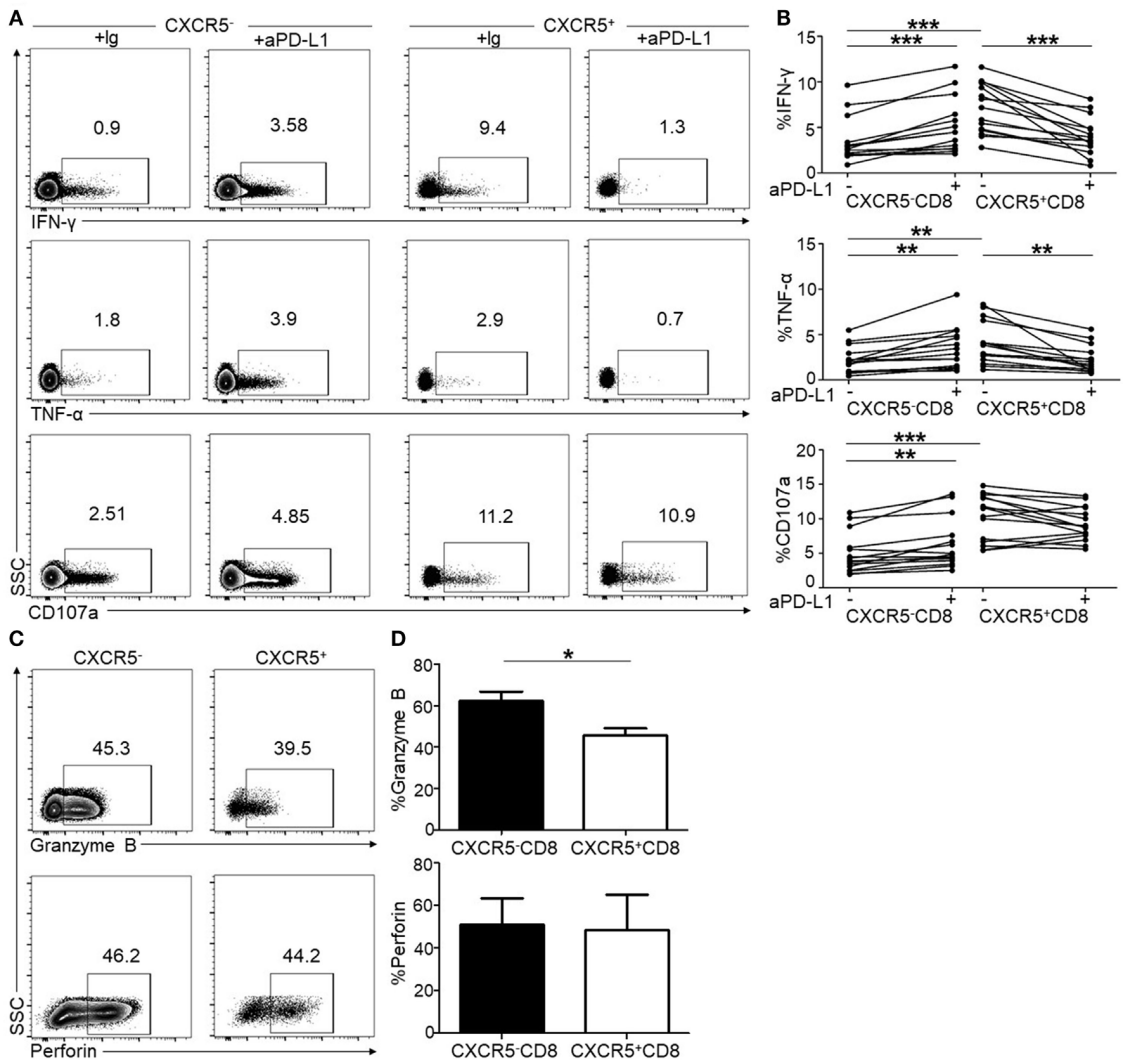
Programmed cell death 1 (PD-1) blockade shows opposite effects on CXCR5^−^ and CXCR5^+^CD8^+^ T cells in peripheral lymphocytes. Peripheral blood mononuclear cells (PBMCs) from HIV-infected patients were stimulated for 8 h using HIV overlapping peptides with or without anti-PD-L1. The flow cytometric plots show intracellular IFN-γ **(A)**, TNF-α **(A)**, and CD107a **(A)** staining on CXCR5^−^ and CXCR5^+^CD8^+^ T cells populations, respectively. **(B)** Statistical analysis of intracellular IFN-γ, TNF-α, and CD107a staining of CXCR5^−^ and CXCR5^+^CD8^+^ T cells (a paired *t-*test was used, **p < 0.01; ***p < 0.001). **(C)** The flow cytometric plots of intracellular granzyme B and perforin. **(D)** Statistical analysis of granzyme B and perforin staining of CXCR5^−^ and CXCR5^+^CD8^+^ T cells (a paired *t-*test was used, *p < 0.05).

Considering a PD-1 pathway blockade as an immunological strategy to restore CTL function during chronic HIV infection (14, 17, 21), we investigated the effect of a PD-1 pathway blockade on both CXCR5^−^ and CXCR5^+^CD8^+^ T cells in response to short-term TCR stimulation *in vitro*. After an 8-h stimulation using HIV-1 derived overlapping peptides with or without anti-PD-L1, the levels of IFN-γ, TNF-α, and CD107a were detected as functional markers of HIV-specific CTL responses. In agreement with previous reports (11), the PD-1 blockade significantly restored the production of IFN-γ and TNF-α (Figures 4A,B) in CXCR5^−^CD8^+^ T cells. Unexpectedly, unlike CXCR5^−^CD8^+^ T cells, the PD-1 pathway blockade inhibited the production of IFN-γ and TNF-α (Figures 4A,B) in CXCR5^+^CD8^+^ T cells, and the statistical analysis revealed a significant difference (Figure 4B). These results indicate that PD-1 expression exhibits opposing functions on CXCR5^−^ and CXCR5^+^CD8^+^ T cells. In addition, CXCR5^+^CD8^+^ T cells produced more IFN-γ and TNF-α than CXCR5^−^CD8^+^ T cells after stimulation using HIV-1 overlapping peptides (Figure 4B). While for CD107a, the PD-1 blockade significantly increased the production of CD107a in CXCR5^−^CD8^+^ T cells, with no significantly changes in CXCR5^+^CD8^+^ T cells (Figures 4A,B). Consistent with previous reports (9), a significant fraction of CXCR5^+^CD8^+^ T cells produced granzyme B and perforin and granzyme B was significantly lower in CXCR5^+^CD8^+^ T cells compared with CXCR5^−^CD8^+^ T cells (Figures 4C,D).

**Figure 5:**
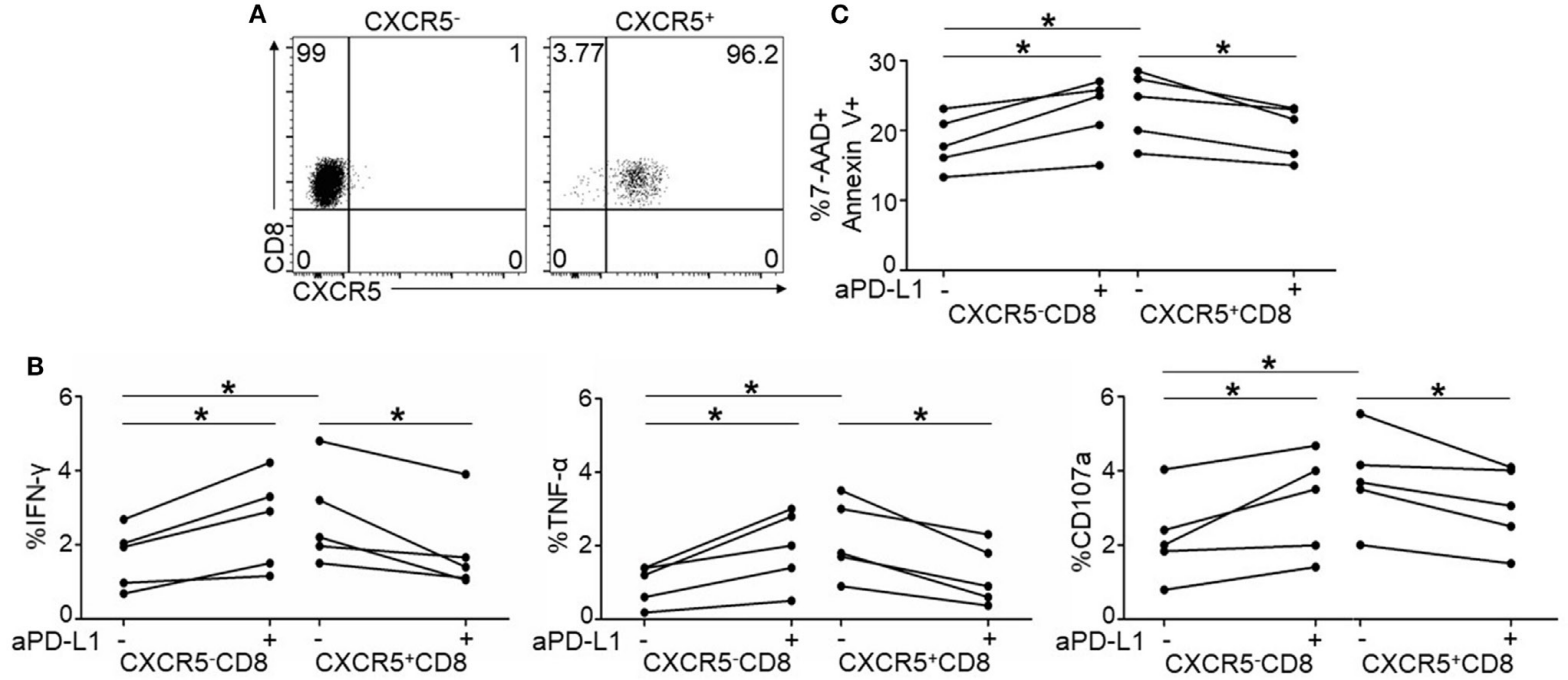
Purified CXCR5^+^CD8^+^ T cells exhibit different response to programmed cell death 1 (PD-1) blockade from CXCR5^−^CD8^+^ T cells. **(A)** Representative flow cytometric data of FACS sorted CXCR5^−^(left) and CXCR5^+^CD8^+^ T cells (right). Data are representative of five individual samples. **(B)** Purified CXCR5^−^ and CXCR5^+^CD8^+^ T cells from HIV-infected patients were stimulated for 8 h using HIV overlapping peptides with or without anti-PD-L1. Statistical analysis of intracellular IFN-γ (left), TNF-α (middle), and CD107a (right) staining on CXCR5^−^ and CXCR5^+^CD8^+^ T cells (*p < 0.05). **(C)** Purified CXCR5^−^ or CXCR5^+^CD8^+^ T cells from HIV-infected patients were co-cultured with Jurkat cells for 8 h with or without anti-PD-L1. Annexin V and 7-AAD staining was performed to show the killing functionality of the cytotoxic T cells (CTLs). Apoptotic Jurkat cells were compared between the different patient groups (*n* = 5 for each group).

To validate the function of PD-1 on CXCR5^+^CD8^+^ T cells, we purified CXCR5^+^ and CXCR5^−^CD8^+^ T cells from the PBMCs. As shown in Figure 5A, the purity of the individual populations was greater than 95%. In response to TCR stimulation, CXCR5^+^CD8^+^ T cells produced higher levels of IFN-γ, TNF-α, and CD107a than CXCR5^-^CD8^+^ T cells (Figure 5B); however, the PD-1 blockade exhibited the opposite effect on CXCR5^+^ and CXCR5^−^CD8^+^ T cells regarding the production of IFN-γ, TNF-α, and CD107a (Figure 5B). This finding was consistent with the data presented in Figure 4. In agreement with cytokine production, the killing capacity of the CXCR5^+^CD8^+^ T cells was greater than the CXCR5^−^CD8^+^ T cells (Figure 5C). The PD-1 blockade exhibited the opposite effect on CXCR5^+^ and CXCR5^−^CD8^+^ T cells (Figure 5C). All these data provide evidence that PD-1^+^CXCR5^+^CD8^+^ T cells could be functional CTLs, rather than an exhausted CTL population during chronic HIV infection.

### Compared CXCR5^+^CD8^+^ T and CXCR5^−^CD8^+^ T Cells between CRs and INRs

**Figure 6:**
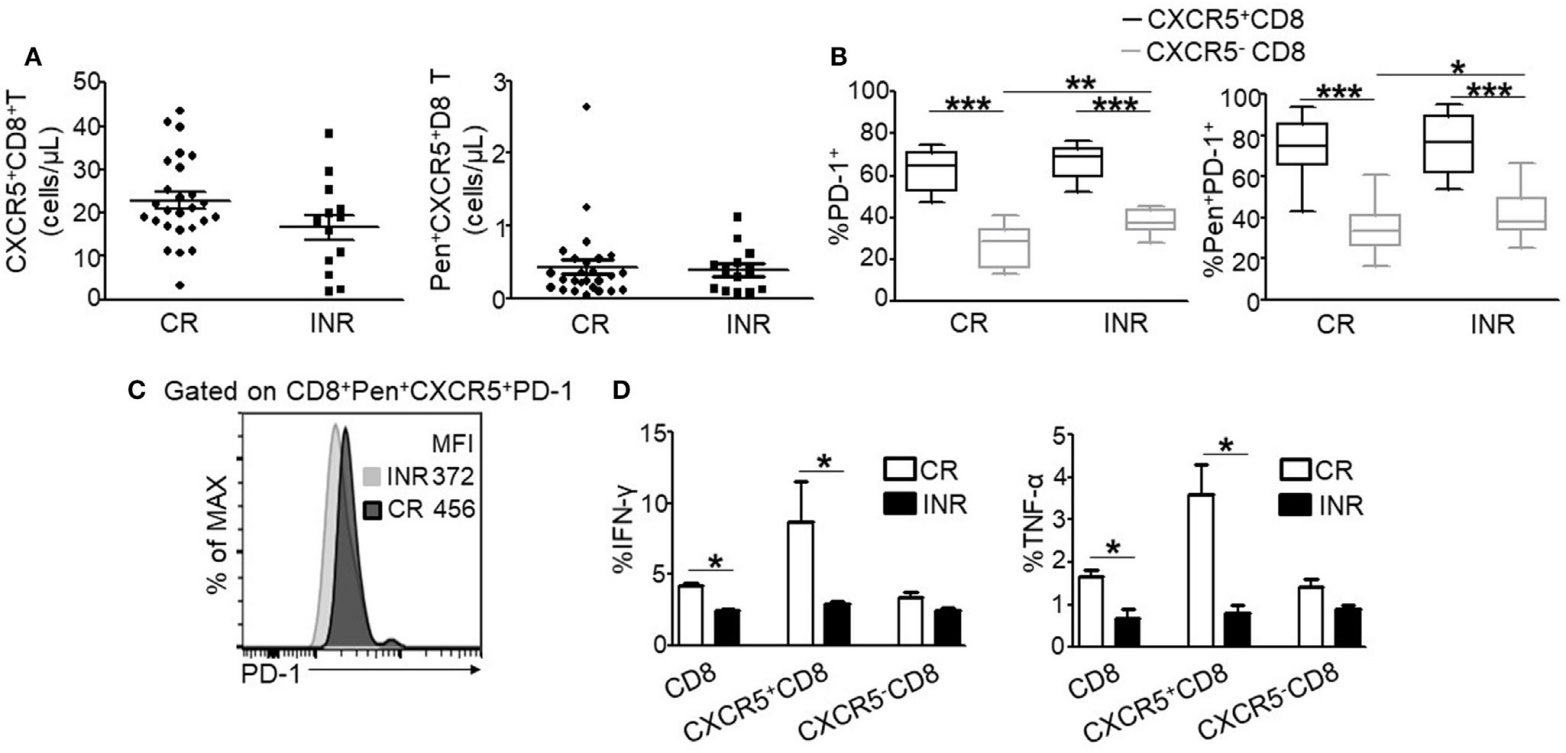
ART treatment fails to restore the function of HIV-specific CXCR5^+^CD8^+^ T cells in immune non-responders (INRs) patients. **(A)** Comparing the number of CXCR5^+^CD8^+^ T cells (left) and Pen^+^CXCR5^+^CD8^+^ T cells (right) between the complete responders (CRs) and INRs. **(B)** Comparison of programmed cell death 1 (PD-1) expression on CXCR5^−^ and CXCR5^+^CD8^+^ T cells gated on the total CD8^+^ (left) and Pentamer^+^ CD8^+^ T cell (right) populations, respectively (*n* = 26 for CRs; *n* = 13 for INRs). **(C)** Representative data of PD-1 mean fluorescence intensity (MFI) on Pentamer^+^CXCR5^+^CD8^+^ T cells from CRs and INRs. The data represent three independent experiments with similar results (*n* > 3 for each group). **(D)** IFN-γ (left) and TNF-α (right) production on the total, CXCR5^−^, or CXCR5^+^CD8^+^ T cells stimulated for 8 h using HIV-derived overlapping peptides *ex vivo* (*n* > 8 for each group). Each dot represents one individual patient. Statistical significance between two groups was determined by a Mann–Whitney non-parametric *U* test.

ART is associated with excellent efficacy for the clearance of peripheral HIV and the resultant immune reconstitution in the CRs. We analyzed CXCR5 expression on circulating total and HIV-specific Pentamer^+^ CTLs after ART. The number of CXCR5^+^CD8^+^ T cells and HIV-specific Pentamer^+^CXCR5^-^CD8^+^ T cells were no statistically significance between CRs and INRs (Figure 6A). Compared to INRs, ART result in the lower expression of PD-1 on total (Figure 6B, left) and HIV-specific Pentamer^+^CXCR5^−^CD8^+^ T cells (Figure 6B, right) in CRs; however, there were no difference of PD-1 expression on CXCR5^+^CD8^+^ T cells between INRs and CRs (Figure 6B). In addition, the fluorescence intensity of PD-1 was higher on CXCR5^+^CD8^+^ T cells in the CRs (Figure 6C). Functionally, the total population of CTLs from the CRs produced more IFN-γ and TNF-α in response to stimulation with HIV-derived overlapping peptides (Figure 6D). Thus, ART retained the quantity of circulating CXCR5^+^CD8^+^ T cells but failed to restore the function of HIV-specific CXCR5^+^CD8^+^ T cells in the INRs.

## Discussion

In this study, we found that HIV-induced CXCR5^+^CD8^+^ T cells correlated with immune control during chronic HIV infection. Unlike CXCR5^−^CD8^+^ T cells which used PD-1 as an exhaustion marker, CXCR5^+^CD8^+^ T cells exclusively retained high PD-1 expression. In addition, high PD-1 expression was associated with CXCR5^+^CD8^+^ T cells functionality. A PD-1 blockade inhibited rather than enhanced the functionality of CXCR5^+^CD8^+^ T cells. Thus, PD-1^+^CXCR5^+^CD8^+^ T cells may be regarded as a functional population during chronic HIV infection.

CXCR5^+^CD8^+^ T cells are induced during chronic HIV or SIV infections (6, 9). In this study, the correlation analysis between CXCR5^+^CD8^+^ T cells and disease progression in TPs revealed that CXCR5^+^CD8^+^ T cells, especially HIV-specific CXCR5^+^CD8^+^ T cells, was positively correlated with peripheral CD4^+^ T cell counts and negatively correlated with the viral load. CXCR5^+^CD8^+^ T cells mainly exist in lymphoid tissues and exhibited strong HIV-specific CTL function. In addition, lymphoid tissue is an important site for HIV replication (35). This may be the reason of negative correlation between CXCR5^+^CD8^+^ T cells and HIV disease progression. These findings indicated that CXCR5^+^CD8^+^ T cells may be involved in disease control and could be used as an immunological marker during a chronic HIV infection.

Moreover, the distribution of CXCR5^+^CD8^+^ T cells in the LNs of HIV-infected patients was analyzed using immunohistochemistry. Although it has been reported that antiviral CD8^+^ T cells have a limited capacity to migrate to the GCs of the lymphoid tissue in infected patients (36–38), we found that CXCR5^+^CD8^+^ T cells were found to be localized in and out of the GCs. Ayala VI (39) demonstrated that engineered CD8 T cells expressing human CXCR5 preferential localization within B-cell follicles. Petrovas et al. (22) observed higher frequencies of CXCR5^hi^CD8^+^ T cells in GCs compared to T cell areas. During chronic SIV infection, SIV-specific CXCR5^+^CD8^+^ T cells have been identified within the extrafollicular and intrafollicular regions of the lymphoid tissue (37). All these results indicate that CXCR5^+^CD8^+^ T cells could enter the GC during HIV infection.

Programmed cell death 1 is considered to be an exhaustion marker expressed on CD8^+^ T cells during chronic infection or tumor progression (10–12, 14–17); however, PD-1 expression on CXCR5^+^CD8^+^ T cells during chronic HIV infection remains poorly defined. In this study, we found that high expression of PD-1 was maintained on CXCR5^+^CD8^+^ T cells and HIV-specific CXCR5^+^CD8^+^ T cells during chronic HIV infection. Moreover, the increased PD-1 expression on CXCR5^+^CD8^+^ T cells was associated with higher CD4^+^ T cell counts. Functionally, the CXCR5^+^CD8^+^ T cells produced more IFN-γ, TNF-α, and CD107a than CXCR5^−^CD8^+^ T cells in response to short-term TCR stimulation *in vitro*. This result is in agreement with other reports (6, 8, 22, 40). These findings indicate that CXCR5^+^CD8^+^ T cells are functional CTLs. Moreover, a blockade of the PD-1 pathway inhibited rather than enhanced the production of IFN-γ and TNF-α by CXCR5^+^CD8^+^ T cells, indicating that PD-1 expression was a functional marker for CXCR5^+^CD8^+^ T cells. Follicular TFHs are characterized by high expression of PD-1 which is also a critical functional molecule for TFHs (23–25). Both TFHs and CXCR5^+^CD8^+^ T cells are main located in lymphoid tissue, CXCR5^+^CD8^+^ T cells in peripheral blood have some common characteristics with CXCR5^+^CD8^+^ T in LNs, whether the microenvironment in lymph tissue leads to the different PD-1 profile between CXCR5^+^CD8^+^ T cells and CXCR5^−^CD8^+^ T cells worth further studying. In addition, Nakamoto et al. also found that HCV-specific CD8 T cells restoration was different in tissues and peripheral blood after PD-1 pathway blocking (41). However, Petrovas et al. (22) observed increased production of IFN-γ and TNF-a from CXCR5^+^CD8^+^ T cells after PD-1 pathway inhibition. There are some differences between Petrovas’s study and this study: differences in patients, tissue, stimulus, and culture conditions. The functional changes of CXCR5^+^CD8^+^ T cells after PD-1 blockade in HIV-infected patients need to be intensive studied in a large number of patients. In addition, in this study, we just detected the effect of blockaded PD-1 for a short time on HIV-specific CXCR5^+^CD8^+^ T cells, it is also an important issue to know the effect of blockaded PD-1for a long time on HIV-specific CXCR5^+^CD8^+^ T cells. In this study, we found that the ability of CXCR5^+^CD8^+^ T cells to produce perforin and granzyme B was lower than that of CXCR5^−^CD8^+^ T cells without PD-1 pathway blockade, and this result is consistent with some previous reports (8, 9, 21). Killing experiment showed that the purified CXCR5^+^CD8^+^ T cells had stronger killing ability to target cells than CXCR5^−^CD8^+^ T cells. Our results showed that the ability of CXCR5^−^CD8^+^ T cells secreting granzyme B was stronger than that of CXCR5^+^CD8^+^ T cells, which suggests that the killing function of CXCR5^+^CD8^+^ T cells may not be *via* granzyme B pathway.

Unlike TFH cells and PD-1^+^CD4^+^ T cells, which are the main HIV reservoirs during ART (38, 42, 43), CD8^+^ T cells are not infected by HIV. Thus, functional PD-1^+^CXCR5^+^CD8^+^ T cells may be utilized to clear the infected target cells in lymphoid tissues. HIV-specific CD8^+^ T cells play an important role in eliminating latent HIV and HIV reservoirs (43). A PD-1 blockade may enhance the function of exhausted CXCR5^−^CD8^+^ T cells; however, PD-1 exhibited the opposite function on CXCR5^+^CD8^+^ T cells in this study. The mechanism of dichotomous functions for PD-1 on CXCR5^+^ and CXCR5^−^CD8^+^ T cell needs further study. Of note, the PD-1 blockade increased the CXCR5^+^CD8^+^ T cell conversion into a CXCR5^−^CD8^+^ T cell (9, 21). Thus, these findings imply that a PD-1 blockade alone may be an inappropriate therapeutic strategy, and it is necessary to maintain the function of CXCR5^+^CD8^+^ T cells as well as the CXCR5^−^CD8^+^ T cell subset, as CD8^+^ T cells expressing CXCR5 are redirected into LN follicles, which are important areas of HIV reservoir.

In this study, we first compared CXCR5^+^CD8^+^ T cells between CRs and INRs. Consistent with previous reports (11, 21), PD-1 expression on CXCR5^−^CD8^+^ T cells in INRs was higher than that in CRs, which was the opposite for CXCR5^+^CD8^+^ T cells. Functionally, HIV-specific CTLs, especially CXCR5^+^CD8^+^ T cells from CRs produced greater levels of IFN-γ and TNF-α in response to HIV peptide stimulation, indicating that ART failed to restore the function of HIV-specific CXCR5^+^CD8^+^ T cells in INRs.

In conclusion, we demonstrated that CXCR5^+^CD8^+^ T cells were negatively associated with HIV disease progression and PD-1^+^CXCR5^+^CD8^+^ T cells were functional CTL population during chronic HIV infection. Furthermore, the functional PD-1^+^CXCR5^+^CD8^+^ T cells failed to be restored in INRs compared to CRs. Our findings indicate that to specifically boost the function of PD-1^+^CXCR5^+^CD8^+^ T cells represent a novel therapeutic strategy for AIDS patients.

## Author Contributions

Y-MJ, H-GY, LY, and F-SW conceived the study, designed the experiments, and analyzed the data; Y-MJ, H-GY, BT, S-JX, and LM performed the experiments; H-HH, BT, S-JX, LM, WX, RH, J-YZ, R-NX, LJ, MS, ZX, E-QQ, X-CW, and HW contributed to reagents and materials; and Y-MJ, H-GY, S-JX, and F-SW wrote the article. All authors read and approved the final manuscript.

## Conflict of Interest Statement

The authors declare that the research was conducted in the absence of any commercial or financial relationships that could be construed as a potential conflict of interest.
